# Endogenous endophthalmitis caused by a multidrug-resistant hypervirulent *Klebsiella pneumoniae* strain belonging to a novel single locus variant of ST23: first case report in China

**DOI:** 10.1186/s12879-018-3543-5

**Published:** 2018-12-17

**Authors:** Min Xu, Ang Li, Haishen Kong, Weili Zhang, Hongchao Chen, Yajie Fu, Yiqi Fu

**Affiliations:** 1 Key Laboratory of Clinical In Vitro Diagnostic Techniques of Zhejiang Province, Center of Clinical Laboratory, the First Affiliated Hospital, College of Medicine, Zhejiang University, Hangzhou, China; 2 Department of Precision Medicine Center, the First Affiliated Hospital of Zhengzhou University, Zhengzhou, China; 3 State Key Laboratory for Diagnosis and Treatment of Infectious Diseases, Collaborative Innovation Center for Diagnosis and Treatment of Infectious Diseases, the First Affiliated Hospital, College of Medicine, Zhejiang University, Hangzhou, China; 4 Department of Respiratory Diseases, the First Affiliated Hospital, College of Medicine, Zhejiang University, Hangzhou, China

**Keywords:** Endophthalmitis, Hypervirulent, *Klebsiella pneumoniae*, AmpC, ESBL

## Abstract

**Background:**

Endogenous endophthalmitis caused by hypervirulent *Klebsiella pneumoniae* (HVKP) is an emerging infectious disease commonly with a devastating visual outcome. Most HVKP strains display a wild-type susceptibility profile to antibiotics. However, reports of antimicrobial-resistant HVKP have increased over time, which poses a serious therapeutic dilemma.

**Case presentation:**

A 25-year-old man with a liver abscess and poorly controlled diabetes mellitus was admitted for endophthalmitis due to *K. pneumoniae*. The isolate displayed hypermucoviscosity as determined by a positive string test and exhibited resistance to ceftazidime, piperacillin-tazobactam and amikacin. Whole genome sequencing (WGS) analysis demonstrated the isolate to be a K1-serotype strain and belong to a novel single locus variant of ST23, ST2922. In addition to the virulence genes linked to HVKP, *rmpA*, *magA*, *iucABCDiutA* (aerobactin), *ybtAPSTUX* (yersiniabactin) and *iroBDN* (salmochelin), it was found to harbor extended-spectrum β-lactamase (ESBL) gene (*bla*_CTX-M-14_), AmpC β-lactamase gene (*bla*_DHA_), and 16S rRNA methylase gene (*armA*).

**Conclusions:**

This is the first known case of endogenous endophthalmitis caused by a multidrug-resistant HVKP strain ever reported in China. Early diagnosis and treatment with intravenous and intravitreal injection of carbapenem were essential for a favorable visual outcome.

## Background

Endophthalmitis is a rare but severe infection that is associated with a poor visual outcome despite early diagnosis and treatment [1]. Over the past two decades, a distinct hypervirulent strain of *Klebsiella pneumoniae* has emerged mainly in Southeast Asia as a clinically significant pathogen responsible for endogenous endophthalmitis secondary to pyogenic liver abscesses (PLA) and diabetes mellitus [2, 3]. This strain is characterized by a hypermucoviscous phenotype determined by the string test. The genomic analysis of hypervirulent *K. pneumoniae* (HVKP) most frequently indicates capsular serotypes K1 and K2, as well as the presence of several important virulence factors including *rmpA* (regulator of mucoid phenotype A), *magA* (mucoviscosity-associated gene A) and siderophores-encoding genes [4]. It is reported that among several siderophore-encoding genes, the genes for aerobactin were more frequently found in HVKP strains [5–7]. Traditionally, most HVKP strains exhibited susceptibility to commonly used antibiotics except for an intrinsic resistance to ampicillin. However, an increase of antimicrobial resistance in HVKP has been observed in recent years. A series of cases including liver abscess, bacteremia and meningitis caused by extended-spectrum β-lactamase (ESBL)-producing HVKP have already been reported [8–10]. Here, we present the first case of endophthalmitis associated with multidrug resistant HVKP coharboring ESBL, AmpC β-lactamase and 16S rRNA methylase genes in China.

## Case presentation

A 25-year-old man, who had a history of diabetes mellitus with poorly controlled blood sugar levels for 7 years, was admitted to a local hospital for fever and right upper quadrant abdominal pain. A computed tomography (CT) scan of his abdomen showed a low-density area of 4.6 cm × 2.9 cm in the caudate lobe of the liver, and he was diagnosed with a liver abscess after magnetic resonance imaging (MRI) with gadolinium enhancement. The patient received intravenous antibiotic without aspiration or drainage of the abscess, and no ophthalmological examination was performed at that time. Detailed information about bacterial cultures and antibiotic treatment in the local hospital was unavailable. One week later, the fever and abdominal pain had resolved. However, visual acuity of the right eye decreased significantly 3 days after relief of the initial symptoms. Therefore, the patient was transferred to our hospital for further ophthalmic examination. On admission, a complete blood cell count (CBC) analysis did not show any change, and a white blood cell (WBC) count of 6700/μl (62.5% segmented neutrophils, 25.6% lymphocytes and 10.9% monocytes) was recorded. However, the blood analysis did reveal an increase in C-reactive protein (60 mg/L) and fasting serum glucose reached 14.53 mmol/L. The patient’s visual acuity in the right eye was light perception only. Slit-lamp examination showed moderate conjunctival injection, corneal infiltrate, along with hypopyon in the affected eye. A presumed diagnose of endophthalmitis was made. Empirical treatment of intravenous imipenem (0.5 g given every 6 h [Q6H]) and intravitreal injection of imipenem, vancomycin and dexamethasone were started immediately. Culture of the vitreous fluid grew a *K. pneumoniae* strain, named KP587. However, blood cultures yielded negative results. Four days later, a pars plana vitrectomy with silicone-oil injection was performed. Imipenem was continued for a total of 16 days and then switched to oral moxifloxacin (0.4 g given daily, [QD]) for another 14 days after discharged. Because symptoms were relieved and the size of the liver abscess was decreasing after antibiotic treatment, percutaneous needle aspiration was not performed based on the judgment of the hepatobiliary surgeon. At a two-week follow-up, the patient’s visual acuity in the affected eye had improved to FC/40 (the patient could count the number of fingers at a distance of 40 cm from his eyes).

### Microbiological analysis

The *K. pneumoniae* strain KP587 demonstrated hypermucoviscosity, as determined by a positive string test. Antibiotic susceptibility testing was conducted using the broth microdilution method. The minimum inhibitory concentrations (MICs) were determined in accordance with the breakpoints defined by the Clinical and Laboratory Standards Institute [11]. For tigecycline, the result was interpreted according to the European Committee on Antimicrobial Susceptibility Testing (EUCAST) criteria (version 7.1, http://www.eucast.org/clinical_breakpoints/). In contrast to the wide-susceptibility profile of most HVKP, this strain was resistant to aztreonam, amikacin, gentamicin, ceftazidime, ceftriaxone and piperacillin-tazobactam, while susceptible to cefepime, imipenem, ciprofloxacin, levofloxacin and tigecycline, as shown in Table 1.

**Table 1:** Antibiotics susceptibility profile of the *K. pneumoniae* strain KP587

Antibiotics	MIC (μg/ml)	Interpretation
Ampicillin	≥ 32	R
Amikacin	≥ 64	R
Aztreonam	≥ 64	R
Ciprofloxacin	0.25	S
Ceftriaxone	≥ 16	R
Gentamicin	≥ 16	R
Levofloxacin	0.5	S
Ceftazidime	16	R
Cefepime	1	S
Piperacillin-tazobactam	≥ 128	R
Ertapenem	≤ 0.5	S
Imipenem	≤ 1	S
Tigecycline	≤ 0.5	S

*MIC* Minimum inhibitory concentration, *I* intermediate, *R* resistant, *S* susceptible

**Fig. 1:**
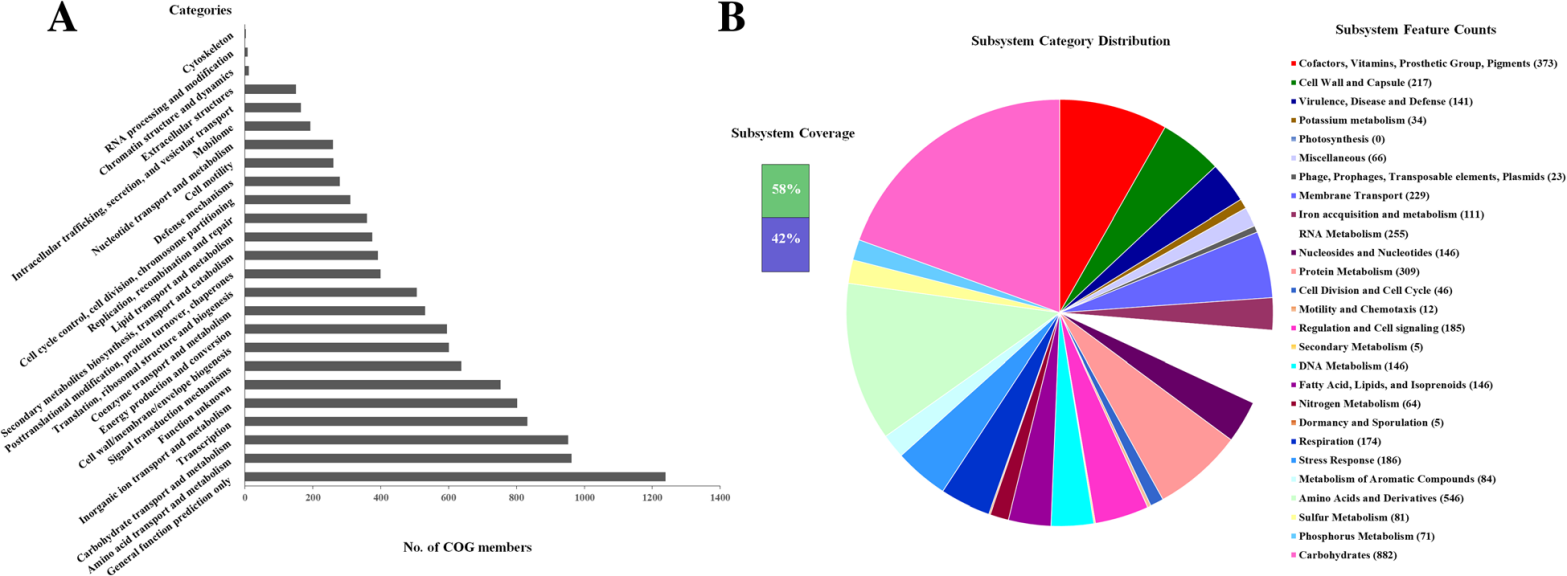
The distribution of genes to COGs categories and SEED subsystems of the *K. pneumoniae* strain KP587. **a** COGs distribution of the *K. pneumoniae*. **b** Distribution of genes assigned to SEED subsystems (based on the RAST annotation server)

Genomic DNA was extracted and sequenced using the HiSeq 2000 platform (Illumina, San Diego, CA, USA) with a 2 × 125 bp paired-end strategy. De novo assembly using Velvet version 1.2.07 produced 108 contigs with an N50 length of 272,305 bp [12]. Using Glimmer version 3.0, 5550 genes including 48 RNA genes and 5502 protein-coding genes were identified [13]. The overall genetic information is listed in Table 2. We then assigned hypothetical or putative functions to 1329 protein-coding genes with the NCBI nucleotide sequence database using NCBI blastn and annotated the genome using the RAST (Rapid Annotation using Subsystem Technology) server [14]. We also categorized 3014 genes into COGs (Clusters of Orthologous Groups of proteins) functional groups [15]. The detailed distribution of genes into COG categories and SEED subsystems for this isolate is shown in Fig. 1. The whole-genome sequence has been deposited in DDBJ/EMBL/GenBank under the accession PETA00000000. The version described in this paper is version PETA01000000.

**Table 2:** General genomic features of the *K. pneumoniae* 587 strain

Element and characteristic	Value
Genome size (bp)	5,811,246
N50 (bp)	272,305
DNA coding region (%)	83.9
DNA G + C content (%)	56.8
Contig numbers	108
Total genes	5550
RNA genes	48
Protein-coding genes	5502
Genes with putative function	1329
Genes assigned to COGs	3014
Genes assigned to Pfam domains	4890
Genes with signal peptides	477
Genes with transmembrane helices	1264

**Fig. 2:**
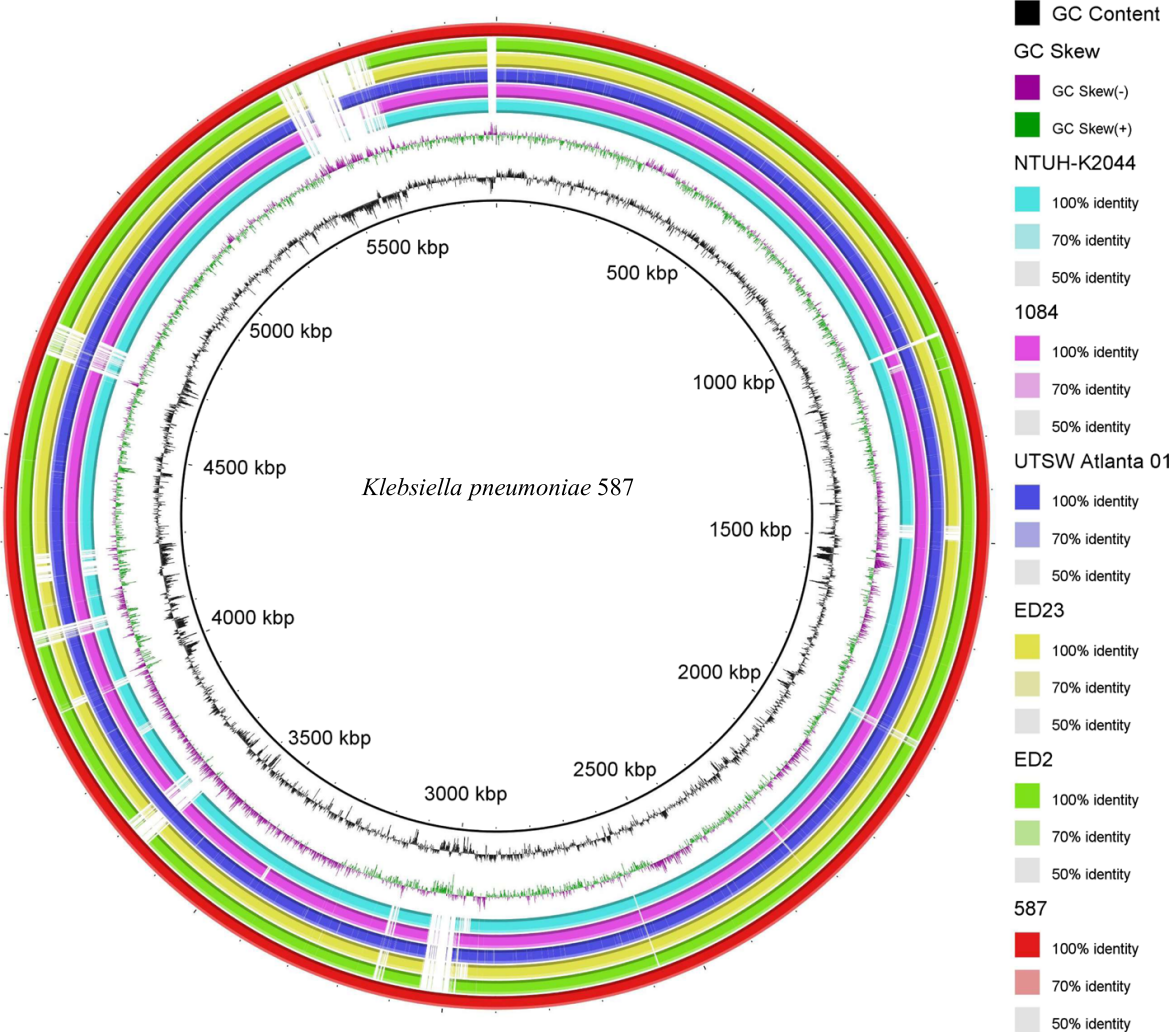
Genomic sequence comparative analysis of *K. pneumoniae* 587 and five serotype K1 *K. pneumoniae* ST23 strains

Analysis of the *wzi* locus indicated that KP587 had a K1 capsular antigen, and 7-gene multilocus sequence typing (MLST) showed that this strain belonged to a novel sequence type, ST2922, a single-locus variant (loci *tonB* with one base difference) of ST23, the most predominant sequence type causing invasive community-acquired infections [16]. Pairwise single nucleotide polymorphisms (SNP) analysis for KP587 and 14 other whole genomic sequences of HVKP available in GenBank using the CSI Phylogeny 1.4 pipeline found that the core genome of KP587 differed from five publicly available ST23 K1-serotype *K. pneumoniae* strains only by a few SNPs (*n* < 100) [17]. We then performed a comparative chromosome sequences analysis of these strains. The circular map generated using BLAST Ring Image Generator (BRIG) showed that KP587 contained several fragments missing in other ST23 stains [18], as shown in Fig. 2. The plasmid replicons of this strain were determined using PlasmidFinder (https://cge.cbs.dtu.dk/services/PlasmidFinder/). IncR, IncFIB and IncHI1B were identified with 95% identity, suggesting the existence of three plasmids. The virulence factors were annotated with the existing database (http://bigsdb.pasteur.fr/klebsiella/klebsiella.html) and the resistant genes were investigated using the web-based tool ResFnder (http://cge.cbs.dtu.dk/services/ResFinder). Together with additional PCR, we found that KP587 harbored *rmpA* and *mag*A genes, which are the main regulators of the mucoid phenotype and are often associated with K1 and K2 capsular types [19]. KP587 possessed the genes coding for siderophores, such as *iucABCDiutA* for aerobactin, *ybtAPSTUX* for yersiniabactin and *iroBDN* genes encoding salmochelin. It also carried the *mrkABCDHIJK* genes encoding type 3 fimbriae. The *allABCDRS* (allantoin utilization) and *kfuABC* (iron acquisition system) gene clusters were present in the genome of this isolate as well. In contrast to most HVKP strains without antibiotic resistance genes, KP587 was found to harbor the ESBL gene *bla*_CTX-M-14_, and the plasmid-mediated AmpC β-lactamase gene, *bla*_DHA_. Additionally, the 16S rRNA methylase gene for aminoglycoside resistance, *armA*, and the plasmid-mediated quinolone resistant gene, *qnrB*, were also detected. Overall, the genomic features of KP587 were consistent with a multidrug resistant hypervirulent strain as previously described [20].

## Discussion and conclusions

Endogenous endophthalmitis is a rare but severe infection that comprises approximately 2–15% of all cases of endophthalmitis [21]. A distinct variant of *K. pneumoniae* characterized by a hypermucoviscous phenotype has been recognized as an important pathogen responsible for endogenous endophthalmitis in some regions of Asian, especially in Taiwan [22] and Korea [23]. Although most HVKP strains displayed susceptibility to most classes of antibiotics, a few cases of infections caused by ESBL-producing HVKP have already been reported [8–10]. However, to the best of our knowledge, this is the first report of endophthalmitis caused by K1-serotype HVKP coharboring AmpC β-lactamase and ESBL genes.

In the present case, the patient was diagnosed with liver abscesses and had a history of poorly controlled diabetes mellitus (hemoglobin A1c was 12.8%). Numerous studies have demonstrated that diabetes is a predisposing risk factor, and liver abscess is a major source of *K. pneumoniae* endophthalmitis [22, 24]. Although percutaneous drainage was not performed and the blood culture was negative, it is still reasonable to believe that the patient in this case suffered from disseminated HVKP infections secondary to liver abscesses. Although previous cases have reported a poor visual outcome [25], appropriate empirical therapy with intravenous and intravitreal imipenem was utilized immediately and the visual outcome of the patient was improved in the current case. Therefore, carbapenem should be the first choice in the treatment of endophthalmitis caused by ESBL-producing HVKP strains.

Many of the virulence genes characteristic of hypervirulent strains were detected in our isolate by whole genome sequencing. It is possible that the number of hypervirulent cases previously reported would be much higher than currently estimated if systematic investigations were performed. Of all the virulence factors, the large virulence plasmid pLVPK-derived loci *rmpA*, *terW*, *iucABCDiutA* and *iroBDN* were detected, which suggested the presence of this plasmid in the strain. Studies have illustrated that *K. pneumoniae* liver abscess formation is associated with the presence of pLVPK [26]. However, the relationship between pLVPK and *K. pneumoniae* endophthalmitis needs further exploration. In our strain, several antibiotic resistant genes were found simultaneously. It is notable that the ESBL gene *bla*_CTX-M-14_, AmpC β-lactamase gene *bla*_DHA-1_, 16S rRNA methylase *armA*, and quinolone resistance gene *qnrB* are usually located on plasmids as well. The genetic exchange between bacteria is a common event. Based on the fact that the majority of HVKP strains possess few antibiotic resistance genes, we speculate that the K1-serotype ST2922 HVKP in our case acquired some resistant plasmids through horizontal transmission, which made the new HVKP strain simultaneously hypervirulent and multidrug resistant. However, no definitive conclusions could be made due to limited information on plasmids, which is a main limitation of our study.

In conclusion, although HVKP endophthalmitis associated with liver abscess is a rare disorder, it is thought to be as a devastating disease. Our case highlighted the need for clinicians to note the diagnosis of HVKP endophthalmitis in patients presenting with ocular symptoms and risk factors. Meanwhile, with the increase of antibiotic resistance in HVKP, attention should be paid to the emergence of multidrug resistant HVKP strains coharboring AmpC β-lactamase and ESBL genes. Early diagnose and treatment with intravenous and intravitreal injection of carbapenems remains as the best approach to improve the visual outcome.

## Abbreviations

CBC: Complete blood cell count
COGs: Clusters of orthologous groups of proteins
CT: Computed tomography
ESBL: Extended-spectrum β-lactamase
HVKP: Hypervirulent *Klebsiella pneumoniae*
MLST: Multilocus sequence typing
MRI: Magnetic resonance imaging
PCR: Polymerase chain reaction
PLA: Pyogenic liver abscesses
pLVPK: Large virulence plasmid of *Klebsiella pneumoniae*
RAST: Rapid annotation using subsystem technology
SNP: Single nucleotide polymorphism
ST: Sequence type
WBC: White blood cell
WGS: Whole genome sequencing
